# Risk Factors for Recurrence in Serous Borderline Ovarian Tumors and Early-Stage Low-Grade Serous Ovarian Carcinoma

**DOI:** 10.3390/curroncol32050263

**Published:** 2025-04-30

**Authors:** Jingjing Zhang, Ming Wang, Yumei Wu

**Affiliations:** 1 Department of Gynecologic Oncology, Beijing Obstetrics and Gynecology Hospital, Capital Medical University, Beijing 100006, China; zjj2018@ccmu.edu.cn (J.Z.); gynoncol111@ccmu.edu.cn (M.W.); 2 Beijing Maternal and Child Health Care Hospital, Beijing 100006, China

**Keywords:** serous borderline ovarian tumors, low-grade serous ovarian carcinoma, recurrence, fertility-sparing surgery, nomogram

## Abstract

Background: Tumor recurrence significantly impacts the quality of life and fertility of patients with serous borderline ovarian tumors (SBOT) and early-stage low-grade serous ovarian carcinoma (LGSOC). This study aims to characterize recurrence patterns, identify independent risk factors for recurrence, and develop a nomogram to predict recurrence-free survival (RFS). Methods: We conducted a retrospective case-control study to investigate recurrence in patients undergoing fertility-sparing surgery (FSS) and radical surgery (RS). Logistic regression and Cox regression were used to identify risk factors. Kaplan–Meier analysis was applied to evaluate RFS. A nomogram was developed based on identified variables to predict RFS. Results: Tumor capsule disruption and micropapillary were associated with higher recurrence risk in the FSS group. Non-invasive implants were associated with higher recurrence risk in the RS group. The nomogram prediction model was developed based on identified risk factors. The area under the curve (AUC) for RFS predictions was 0.74 (95% CI: 0.62–0.85) at 3 years and 0.78 (95% CI: 0.67–0.89) at 5 years for the FSS group and 0.87 (95% CI: 0.76–0.98) at 3 years and 0.81 (95% CI: 0.65–0.97) at 5 years for the RS group. Conclusions: We identified the risk factors for recurrence of SBOT and early-stage LGSOC following FSS and RS procedures and developed a predictive model for forecasting RFS. This model provides valuable guidance for patients and clinicians in predicting recurrence risk for patients.

## 1. Introduction

Epithelial ovarian cancer (EOC) is one of the most lethal malignancies of the female reproductive system. Serous EOC represents the most common histological subtype, accounting for approximately 75% of cases, according to the fifth edition of the World Health Organization (WHO) classification of female genital tumors. Historically, EOC was considered a homogeneous entity, with therapeutic strategies primarily based on staging and grading. However, advances in research have shed light on the fact that different pathological types and molecular subtypes of EOC have distinct treatment strategies, outcomes, and prognoses.

According to the “dualistic model” proposed by Kurman and Shih [1,2], EOC is categorized into two main groups: type I and type II tumors, which correspond to distinct tumorigenic pathways. Type I tumors are typically low-grade neoplasms that develop gradually from borderline tumors, whereas type II tumors are high-grade neoplasms that develop de novo, without identifiable precursor lesions. Low-grade serous ovarian carcinoma (LGSOC) is the prototypical type I tumor, while high-grade serous ovarian carcinoma (HGSOC) represents the typical type II tumor.

A significant proportion of LGSOCs are thought to arise in association with serous borderline ovarian tumors (SBOT). Histopathological studies have demonstrated that more than 75% of noninvasive micropapillary serous carcinoma (MPSC) cases exhibit progressive transformation from adenofibromas or atypical proliferative serous tumors. Additionally, over 60% of invasive MPSC cases are associated with noninvasive MPSC, atypical proliferative serous tumors, or adenofibromas, alone or in combination [3]. Genetically, KRAS or BRAF mutations are frequently observed in SBOT and LGSOC [4,5,6]. These findings suggest a morphological and genetic continuum between SBOT and LGSOC, beginning with benign serous cystadenoma/adenofibroma and culminating in LGSOC. In contrast, HGSOC is predominantly characterized by TP53 mutations, homologous recombination repair defects, and chromosomal instability.

From a clinical perspective, compared to the highly invasive and aggressive nature of HGSOC, SBOT and LGSOC are considered low-malignancy tumors. These tumors typically occur at a younger age and have a more favorable prognosis. The 5-year overall survival rates for borderline ovarian tumors (BOT) and LGSOC exceed 90% and 65%, respectively [7,8]. Despite their favorable prognosis, recurrence rates remain a concern, with 11–54.6% of BOT cases and 49.5% of LGSOC cases experiencing at least one recurrence after the primary surgery [9,10].

Previous studies have identified factors such as International Federation of Gynecology and Obstetrics (FIGO) stage, age at diagnosis, gross residual disease (RD), microinvasion, and lymph node involvement as predictors of recurrence in SBOT and LGSOC [9,11,12]. Advances in treatment have introduced fertility-sparing surgery (FSS) as an option for patients with SBOT and early-stage LGSOC, following National Comprehensive Cancer Network (NCCN) guidelines. However, FSS is associated with an increased risk of recurrence [10]. The prognostic determinants influencing disease recurrence in SBOT and early-stage LGSOC patients undergoing FSS or radical surgery (RS) remain insufficiently characterized in current clinical research. While several studies have investigated predictors of recurrence, there is limited research on recurrence patterns and the development of reliable multivariate models for predicting recurrence risk in SBOT and early-stage LGSOC. Therefore, this study aimed to describe recurrence patterns, including recurrence site, detection methods, and changes in pathological type; identify independent risk factors for postoperative recurrence; and establish a nomogram to predict recurrence-free survival (RFS) of SBOT and early-stage LGSOC undergoing FSS.

## 2. Materials and Methods

A total of 118 patients with SBOT and FIGO stage I LGSOC, who underwent primary surgical treatment at Beijing Obstetrics and Gynecology Hospital, Capital Medical University (Beijing, China), between January 2009 and December 2021, were enrolled in this study. Patients were stratified into the FSS group and the RS group based on primary surgical approach, with longitudinal monitoring of post-treatment recurrence state. The selection criteria for FSS were determined through multidisciplinary evaluation incorporating established guidelines [13] and patients’ fertility preservation requirements. FSS involved preserving at least one ovary and the uterus and included procedures such as unilateral ovarian cystectomy (UC), bilateral ovarian cystectomy (BC), unilateral salpingo-oophorectomy (USO), or unilateral salpingo-oophorectomy with contralateral ovarian cystectomy (USO + CC).

Pathology results from the primary surgeries were independently and blindly reviewed by two pathologists and confirmed as SBOT/LGSOC based on the 2020 World Health Organization (WHO) Classification of Tumors of Female Reproductive Organs. Patients with a history of other primary malignancies within five years or incomplete clinical data were excluded. This study was approved by the Ethics Committee of Beijing Obstetrics and Gynecology Hospital, Capital Medical University (Approval Number: IEC-C-29-V03-FJ1).

Demographic, surgical, pathological, and follow-up data were collected from the hospital’s electronic medical record system by two researchers. According to the NCCN guidelines, the standard surgical procedure includes total hysterectomy with bilateral salpingo-oophorectomy (TH + BSO), multiple peritoneal biopsies, removal of pelvic lesions, omentectomy, and lymphadenectomy. Any deviation from this standard procedure was defined as incomplete staging. Tumor staging was determined using the FIGO 2014 criteria. For cases with incomplete staging, tumor stage was assessed based on surgical and pathological records.

According to the 2020 WHO Classification of Tumors of the Female Reproductive Organs, the tumors in this study were classified as follows: (1) classical SBOT; (2) MPSC, defined as tumors with a micropapillary or cribriform growth area >5 mm; (3) SBOT with microinvasion, where stromal invasion is <5 mm in greatest dimension at any single focus; (4) microinvasive LGSOC, where the tumor exhibits LGSOC morphology with invasion <5 mm in greatest dimension; (5) LGSOC. High-risk pathological features, including micropapillary structures, microinvasion, extraovarian noninvasive implants, and extraovarian invasive implants, were collected and analyzed in this study.

Follow-up was conducted through outpatient visits or telephone calls. The following data were collected after primary surgery: general condition, physical examination findings, serum tumor markers (CA125), and imaging results. Recurrence was diagnosed based on a comprehensive evaluation of newly presented symptoms, radiological findings, and CA125 levels and was considered when any of the following occurred: (1) progressive elevation of CA125; (2) detection of a mass on physical examination or new positive imaging findings; (3) pleural effusion or ascites; (4) unexplained intestinal obstruction unresponsive to conservative treatment. RFS was defined as the period from the date of surgery or last chemotherapy to the date of the first recurrence or the last follow-up. The follow-up cutoff date was March 20, 2025. Pathological upgrading was defined as a change to a more aggressive pathology after recurrence, as follows: (1) classical SBOT before recurrence, followed by MPSC, SBOT with microinvasion, microinvasive LGSOC, or HGSOC after recurrence; (2) SBOT with microinvasion before recurrence, followed by MPSC or LGSOC after recurrence; (3) MPSC before recurrence, followed by LGSOC after recurrence. Any deviation from this pattern was considered pathological downgrading.

Statistical analysis was performed using R software (version 4.4.2) and the online platform Zstats (www.medsta.cn/software). Enumeration data were analyzed using chi-square or Fisher’s exact test and expressed as percentages. Measurement data were analyzed using Student’s t-test or non-parametric tests and presented as the mean ± standard deviation (SD) or median with range. Univariate and multivariate logistic regression analyses were conducted to identify independent risk factors for recurrence. Independent risk factors for RFS were evaluated using a Cox proportional hazards model. Kaplan–Meier survival curves were generated for survival analysis. Based on these results, a nomogram for recurrence prediction was developed using R software. Harrell’s concordance index (C-index) was calculated to assess the model’s ability to distinguish between observed and predicted outcomes, with a higher C-index indicating better discriminative ability. The receiver operating characteristic (ROC) curve and area under the curve (AUC) were used to evaluate the model’s sensitivity and specificity. Model performance was validated using the bootstrap method with 1000 internal resampling iterations. Additionally, calibration curves and decision curve analysis (DCA) were employed to assess the clinical utility of the nomogram. Total points were calculated for all patients, and the “survminer” package was used to determine the optimal cutoff value for total points, classifying patients into low- and high-risk subgroups. Kaplan–Meier analysis with the log-rank test was conducted to compare survival differences between the two subgroups. All tests were two-sided, with a significance level set at 5%. *p*-values < 0.05 were considered statistically significant.

## 3. Results

### 3.1. Patterns of Recurrence for FSS Group and RS Group

A total of 66 patients were included in the FSS group and 52 patients in the RS group based on the inclusion and exclusion criteria. In the FSS group, disease recurrence was observed in 54 patients (81.8%), demonstrating a median RFS of 53.5 months (range: 6–156 months). Among the 12 recurrence-free patients, the median RFS was 79 months (range: 52–137 months). In contrast, the RS group exhibited recurrence in 14 cases (26.9%) with a median RFS of 75.5 months (range: 8–130 months), while 38 patients (73.1%) remained disease-free with a median RFS of 76 months (range: 49–145 months).

The forms of first recurrence, including imaging recurrence, biochemical recurrence, and clinical recurrence, were summarized in Table 1. In the FSS group, imaging-confirmed recurrence was documented in 94.4% (51/54) of recurrent cases, while biochemical recurrence (CA125 elevation) occurred in 53.7% (29/54). Symptomatic presentations (abdominal distension/pain, menstrual irregularities) manifested in only 14.8% (8/54). In contrast, the RS group demonstrated universal radiologic evidence of recurrence (14/14, 100%) with near-complete biochemical recurrence (92.9%, 13/14). Clinically apparent disease was rare, observed in merely 2 cases (14.3%).

Table 2 outlines the recurrence locations and frequencies among the two groups. Recurrence patterns demonstrated consistent localization tendencies across both groups. Localized recurrences predominated in both the FSS group (47/54, 87.0%) and RS group (10/14, 71.4%). Disseminated recurrences (pelvic, abdominal, or multisite involvement) were significantly less frequent, accounting for only 13.0% (7/54) and 28.6% (4/14), respectively.

Table 3 and Table 4 comprehensively outline the histopathological outcomes following secondary surgeries in recurrent cases across both treatment groups. In the FSS group (Table 3), 40 of 54 (74.1%) recurrent patients underwent reoperation, with postoperative pathology revealing concordant histological findings with initial diagnoses in 22 cases (55.0%), pathological downgrading in 9 cases (22.5%), and upgraded tumors in 9 cases (22.5%). Comparatively, the RS group (Table 4) documented 8 (57.1%) reoperated cases among 14 recurrences, demonstrating histopathological consistency with primary surgical specimens in 4 cases (50.0%), evidence of pathological regression in 1 case (12.5%), and disease progression in 3 cases (37.5%).

### 3.2. Patients Clinicopathological Characteristics

The clinicopathological characteristics of the FSS and RS groups, including age at diagnosis, CA125 levels, pathological types, FIGO stage, tumor size, surgical methods, FSS, and chemotherapy, were summarized in Table 5 and Table 6, respectively. The comparative analysis of patients with recurrence (*n* = 54) and non-recurrence (*n* = 12) within the FSS group reveals several significant findings in Table 5. Notably, age distribution showed that 90.7% of patients in the recurrence group were younger than 40 years, compared to 75% in the non-recurrence group; however, this difference did not reach statistical significance. Tumor laterality also demonstrated no statistically meaningful distinction (unilateral: 59.3% vs. 83.3%; *p* = 0.186). A pronounced discrepancy was observed in tumor capsule disruption, with significantly higher rates in the recurrence group (72.2%) compared to non-recurrence (25.0%; *p* = 0.006). FIGO stage I patients were predominant in both groups (85.2% vs. 100%), though this difference was not statistically significant. The presence of micropapillary features exhibited a highly significant statistical disparity between the two groups, with occurrences at 70.4% in the recurrence group compared to 16.7% in the non-recurrence group (*p* = 0.001). Surgical approaches demonstrated a slight preference for laparoscopy in the recurrence group (63.0%) versus laparotomy in the non-recurrence group (66.7%; *p* = 0.060). Finally, while adjuvant chemotherapy was exclusively administered to the recurrence group (7.4%), its application did not yield statistically significant differences across study parameters.

In the RS group, certain clinicopathological features showed significant differences between the recurrence (*n* = 14) and non-recurrence groups (*n* = 38) in Table 6. The FIGO stage distribution displayed a marked difference, with stage I being more prevalent in the non-recurrence group (78.9%) compared to the recurrence group (35.7%; *p* = 0.006). Additionally, micropapillary features were notably more common in the recurrence group (92.9% vs. 55.3%; *p* = 0.019). Tumor capsule disruption was significantly associated with recurrence, being present in 92.9% of cases versus 60.5% (*p* = 0.040). Non-invasive implants were more frequently found in the recurrence group, with statistical significance (*p* = 0.005). Other factors like age, CA125 levels, and surgical approach showed no significant difference between the groups.

The correlation between various clinical indicators and recurrence was further analyzed. Results from univariate and multivariate logistic regression analyses for recurrence in the FSS group and the RS group are presented in Table 7 and Table 8, respectively. Univariate and multivariate analysis identified tumor capsule disruption and micropapillary features as independent risk factors for recurrence in the FSS group, while non-invasive implants were significant in RS group.

**Figure 1:**
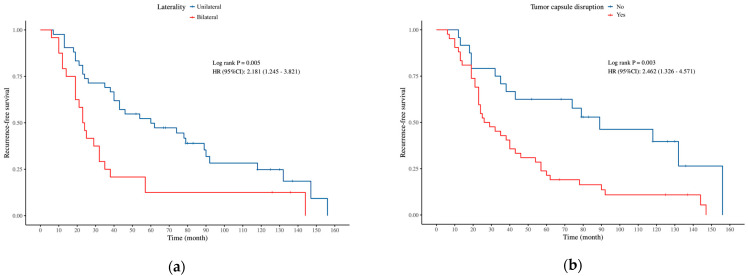
Kaplan–Meier survival analysis of 2 independent prognostic factors of RFS for FSS group: (**a**) laterality, (**b**) tumor capsule disruption.

**Figure 2:**
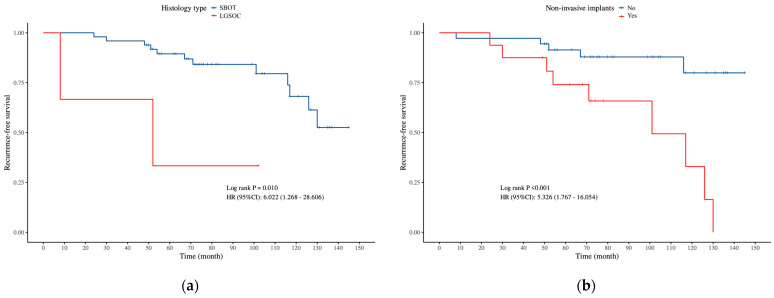
Kaplan–Meier survival analysis of 2 independent prognostic factors of RFS for RS group: (**a**) histology type, (**b**) non-invasive implants.

To further examine the association between various prognostic variables and RFS in the FSS and RS groups, univariate and multivariate Cox proportional hazards regression models were applied, with results shown in Table 9 and Table 10. Univariate and multivariate analysis identified laterality and tumor capsule disruption as independent risk factors for RFS in the FSS group, while histology type and non-invasive implants were independent factors in the RS group. Kaplan–Meier survival analysis, consistent with the Cox regression findings, also revealed that these variables were significantly associated with RFS (Figure 1 and Figure 2).

### 3.3. Development and Validation of the Nomogram

**Figure 3:**
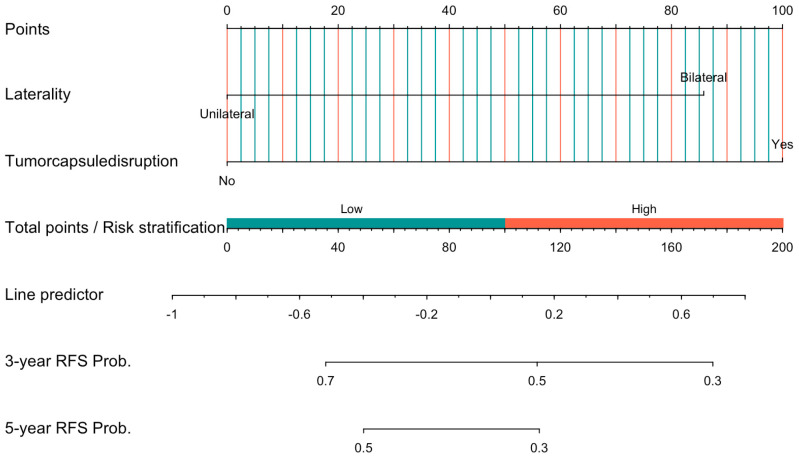
Nomogram and risk stratification for predicting 3- and 5-year RFS probability of patients in FSS group.

**Figure 4:**
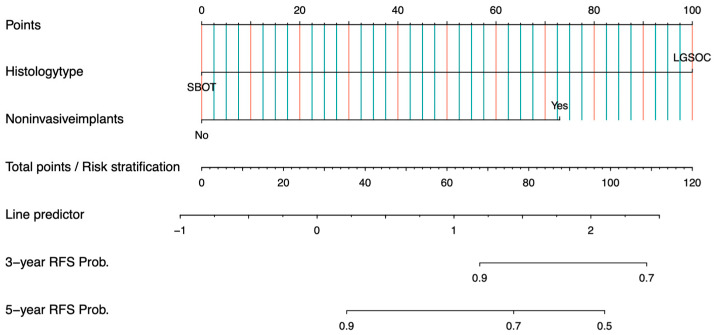
Nomogram and risk stratification for predicting 3- and 5-year RFS probability of patients in RS group.

**Figure 5:**
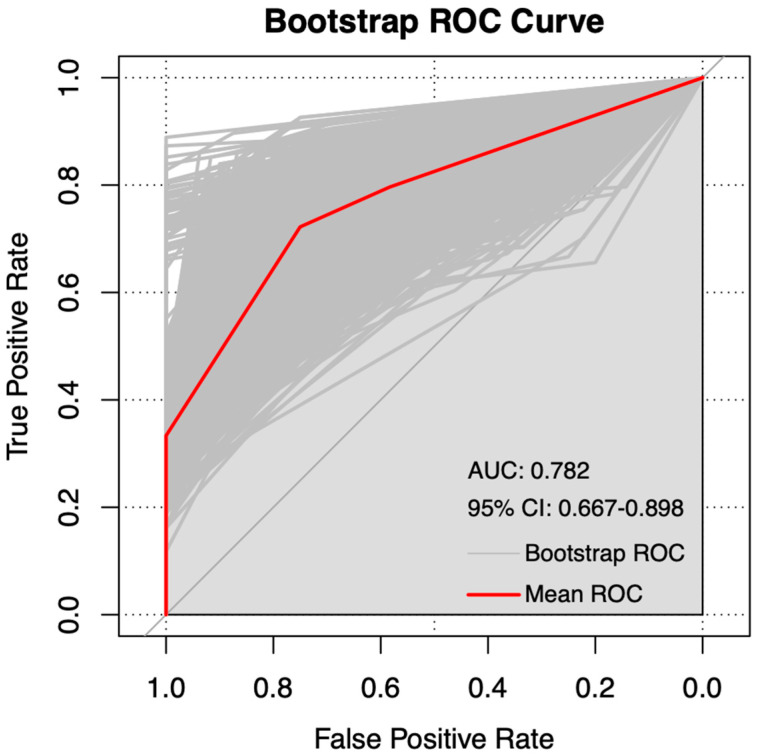
Internal validation and the ROC using the bootstrap method with 1000 resampling iterations in FSS group.

**Figure 6:**
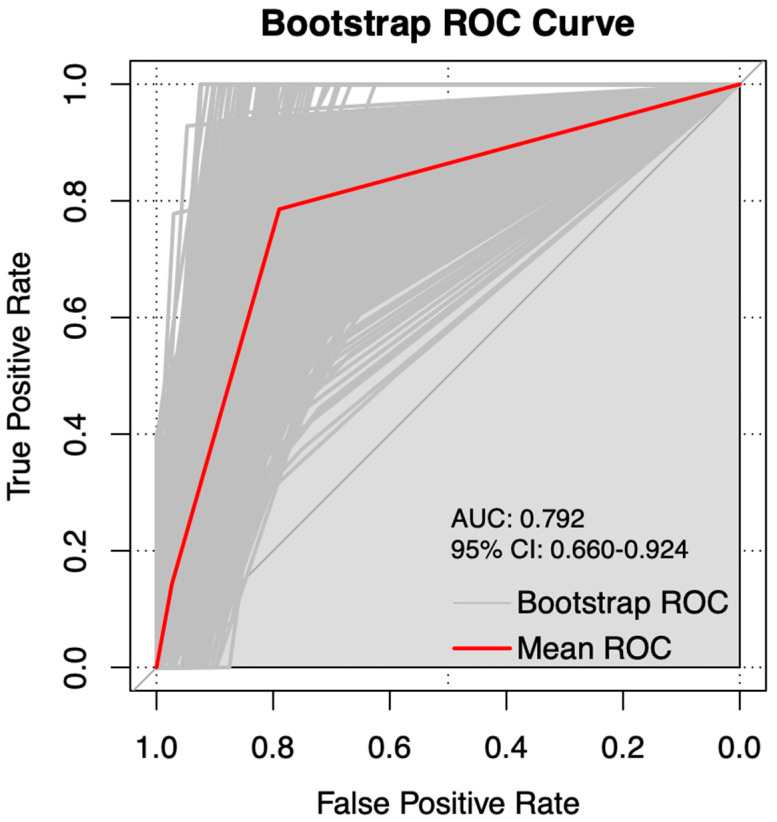
Internal validation and the ROC using the bootstrap method with 1000 resampling iterations in RS group.

Based on the independent prognostic factors identified through Cox regression analysis, we developed a nomogram to predict 3- and 5-year RFS for the FSS group (Figure 3) and the RS group (Figure 4). Internal validation was performed using the bootstrap method with 1000 resampling iterations, resulting in an AUC of 0.7823 (95% CI: 0.667–0.898) in the FSS group (Figure 5) and an AUC of 0.792 (95% Cl: 0.660–0.924) in the RS group (Figure 6).

**Figure 7:**
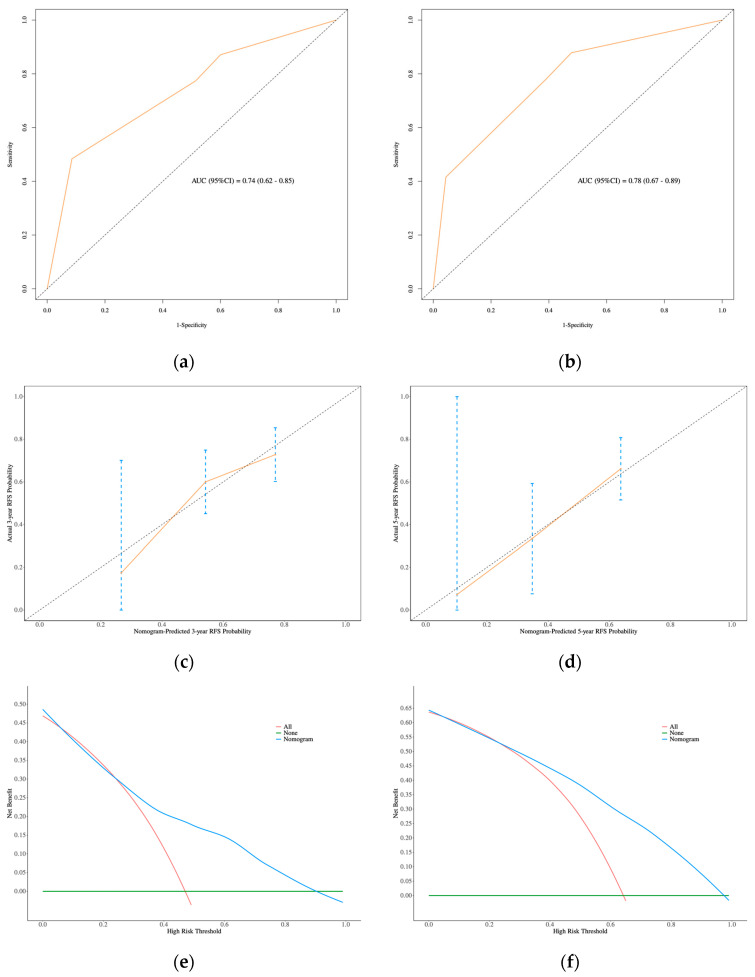
In FSS group: (**a**) and (**b**): ROC curve of nomogram for 3- and 5-year RFS prediction. (**c**) and (**d**): The calibration curves of 3- and 5-year RFS prediction. (**e**) and (**f**): The DCA for 3- and 5-year RFS prediction.

Additionally, in the FSS group, the ROC curve revealed AUC values of 0.74 (95% CI: 0.62–0.85) at 3 years and 0.78 (95% CI: 0.67–0.89) at 5 years, demonstrating the model’s relatively strong discriminatory power (Figure 7a,b). The calibration curves for 3- and 5-year RFS in the FSS group, which closely aligned with the diagonal line, further suggested that the model provided accurate predictions (Figure 7c,d). Finally, decision curve analysis (DCA) was performed to assess the clinical net benefit, which confirmed the nomogram’s high clinical utility (Figure 7e,f), indicating that the model is generalizable for clinical practice.

**Figure 8:**
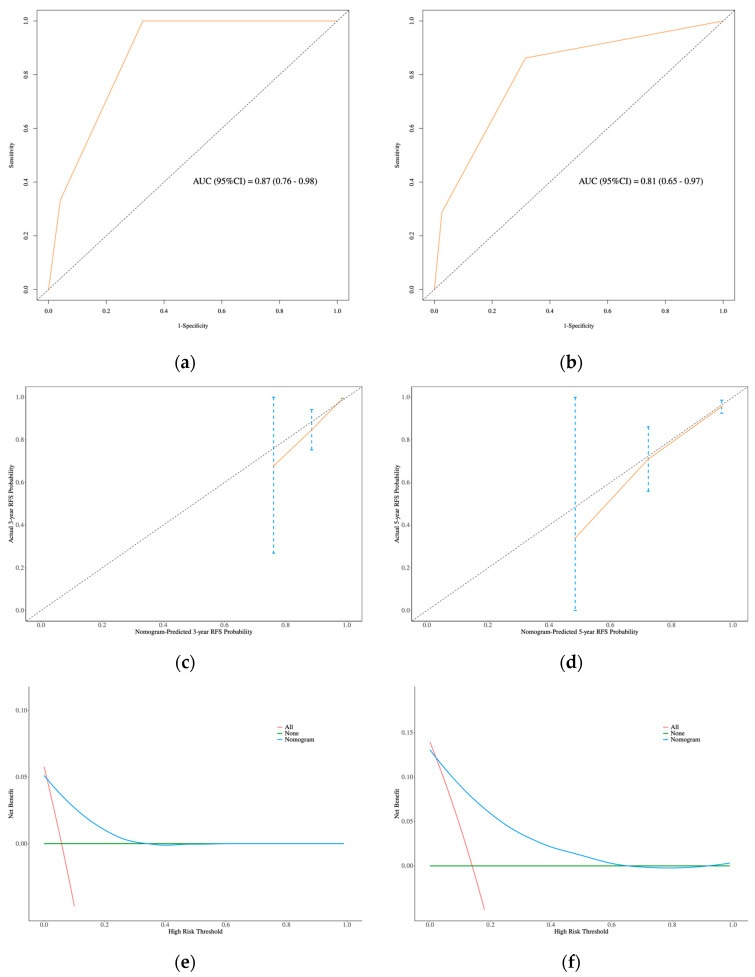
In RS group: (**a**) and (**b**): ROC curve of nomogram for 3- and 5-year RFS prediction. (**c**) and (**d**): The calibration curves of 3- and 5-year RFS prediction. (**e**) and (**f**): The DCA for 3- and 5-year RFS prediction.

In the RS group, the ROC curve revealed AUC values of 0.87 (95% CI: 0.76–0.98) at 3 years and 0.81 (95% CI: 0.65–0.97) at 5 years, demonstrating the model’s relatively strong discriminatory power (Figure 8a,b). The calibration curves for 3- and 5-year RFS in the RS group, which closely aligned with the diagonal line, further suggested that the model provided accurate predictions (Figure 8c,d). DCA was performed to assess the clinical net benefit, which confirmed the nomogram’s high clinical utility (Figure 8e,f), indicating that the model is generalizable for clinical practice.

## 4. Discussion

BOT and LGSOC are rare epithelial ovarian cancers (EOC), accounting for less than 10% and approximately 5% of all EOCs, respectively [14,15]. SBOT, the most common BOT subtype, comprises 50% to 55% of BOT cases. These tumors predominantly affect younger women, with SBOT typically diagnosed between 38 and 42 years of age [5,16] and LGSOC between 43 and 56 years [7,9,17]. Both are frequently associated with infertility, likely due to their ovarian involvement and treatments that impair reproductive function. Despite their favorable prognosis compared to HGSOC, SBOT and LGSOC pose a significant risk of recurrence, with rates ranging from 2–24% for SBOT [18] and over 50% for LGSOC. These tumors share overlapping pathological features and biological behaviors, making differentiation challenging and contributing to a recurrence burden that impacts patient quality of life and healthcare costs, and may exhibit distinct recurrence patterns and risk factors from HGSOC.

Our study aimed to evaluate the recurrence patterns of SBOT and early-stage LGSOC undergoing FSS and RS by comparing clinical data from recurrent and non-recurrent patients. We identified significant differences between these two treatment methods in terms of demographic, clinical, and pathological characteristics, highlighting key factors that influence recurrence risk. For patients with SBOT and early-stage LGSOC, FSS serves as a crucial component of their treatment strategy; however, FSS also contributes to elevated recurrence rates after surgery [19]. In our study, the recurrence rate in the FSS group was 81.8% (54/66) compared to 26.9% (14/52) in the RS group. The existing literature suggests that the recurrence rate of BOT following FSS typically ranges from 5% to 34% [20,21,22]. The unusually high recurrence rate observed in this study is probably attributed to selection bias inherent in the case-control study design at the time of patient enrollment.

In this research, we applied two models—the logistic regression model and the Cox proportional hazards model—to evaluate the recurrence risk factors of SBOT and early-stage LGSOC. In the clinical context, the differences between the two models are significant due to their distinct definitions of study endpoints. The logistic regression model typically evaluates whether an event occurs, functioning as a binary classification model. In our study, micropapillary features and tumor capsule disruption were identified as important risk factors. These characteristics reflect the “probability” of tumor recurrence, indicating whether a patient will experience recurrence within a specific timeframe. On the other hand, the Cox proportional hazards model assesses the time to event (RFS). While it considers whether an event occurs, it also evaluates the rate or timing pattern of the event. Therefore, the results from this model may better capture the long-term biological behavior of tumors, such as how certain factors influence the implicit pace of tumor progression. In our analysis, micropapillary features were potentially associated with the likelihood of recurrence, while tumor laterality might be related to the potential postoperative residual lesions, thereby significantly impacting RFS. Interestingly, capsule disruption was a significant factor in both models. This suggests that capsule disruption influences not only the direct occurrence of recurrence but also the timing or progression dynamics of tumor recurrence, making it a critical prognostic indicator across different analytic frameworks.

Moreover, there remains some debate among studies regarding the high-risk factors affecting the prognosis of patients with BOT undergoing FSS. Presently, the high-risk factors influencing the prognosis and recurrence of advanced BOT patients are summarized as follows: the presence of microinvasion, micropapillary subtype, and peritoneal implants (particularly invasive implants) [23]. Our multivariate analysis identified tumor capsule disruption (HR = 2.369, *p* = 0.007) and bilateral involvement (HR = 2.075, *p* = 0.011) as FSS-specific risk factors, aligning with Hannibal et al.’s finding that bilateral tumors increase invasive recurrence risk by 7.7-fold [24]. The capsule disruption phenomenon may facilitate microscopic seeding, explaining why 87% of FSS recurrences localized to residual ovarian tissue. This mirrors Uzan et al.’s observation that ovarian conservation triples recurrence risk compared to adnexectomy [10]. Notably, RS patients exhibited distinct risk profiles, with non-invasive implants (HR = 8.840, *p* = 0.001) and LGSOC histology (HR = 19.809, *p* = 0.002) driving recurrence. LGSOC type, considered an independent recurrence risk factor, influences the recurrence of disease in patients with RS, possibly due to its inherently more aggressive biological behavior compared to SBOT. Adjuvant chemotherapy did not significantly alter recurrence risk in our study, aligning with the known limited chemosensitivity of SBOT and LGSOC. Surgical approach did not exert a significant impact on recurrence either. Non-invasive implants often evade detection during initial surgery, only to become sources of future disease. Pathological evaluations frequently miss small foci of non-invasive implants, necessitating extensive sampling techniques as advised by Colombo et al. [25]. Moreover, invasive implants are a crucial adverse prognostic factor for patients with serous borderline ovarian tumors (SBOT). An analysis conducted by Morice et al. encompassing seven related studies with a total of 458 advanced SBOT patients revealed that those with invasive implants in stage II-III experienced invasive recurrence and mortality rates of 29% and 25%, respectively, which are significantly higher than the 8.3% and 3.6% observed in patients with non-invasive implants [26]. Additionally, a study by Seidman et al., which included 4129 SBOT patients with a median follow-up period of 7.4 years, demonstrated a survival rate of 95% for patients with non-invasive implants, compared to only 66% for those with invasive implants [27]. In our study, we did not identify invasive implants as independent risk factors for recurrence, potentially due to the limited number of patients with such implants. An expanded sample size is required to achieve more comprehensive and conclusive results. Several studies have highlighted other key factors influencing recurrence risk in SBOT and LGSOC patients, such as surgical approach, chemotherapy after surgery, and pathological characteristics [28]. Micropapillary patterns have also been identified as significant predictors of recurrence [29]. Additionally, research indicates that SBOT patients have a higher recurrence risk following conservative management [30], and recurrence is linked to non-invasive implants and advanced disease stage [16]. Due to the limited sample size, our study was unable to reach any statistical conclusions on pathological characteristics and FIGO stage.

Additionally, our study developed a nomogram based on Cox regression analysis, incorporating these independent prognostic factors, to predict RFS for SBOT and LGSOC patients. The nomogram demonstrated excellent discriminatory ability, with a high C-index and AUC, suggesting its potential utility in clinical practice for identifying patients at higher risk of recurrence. Such tools could significantly contribute to personalized treatment strategies, allowing for better surveillance and intervention. Routine use of this nomogram requires external validation.

This study is subject to several limitations. Primarily, its retrospective nature may engender information bias, influencing the fidelity of data collection and recording, thereby potentially compromising the accuracy of the results [31]. Secondarily, being conducted at a single research center with a limited sample size could hinder the broad applicability of its outcomes, as findings from such studies might not encapsulate the diversity observed in larger or multicenter populations. Future endeavors should prioritize validating these findings through multicenter cohorts to confirm their generalizability and further refine predictive models that incorporate molecular and genetic data. This would not only enhance the prognostic accuracy of such models but could also lead to novel therapeutic strategies tailored to the specific recurrence patterns of SBOT and LGSOC, ultimately improving patient survival and quality of life.

## 5. Conclusions

In summary, SBOT and early-stage LGSOC share similar recurrence patterns, with a high recurrence rate that significantly affects fertility outcomes. Our study evaluated the recurrence patterns of FSS and RS patients and analyzed data from 118 recurrent and non-recurrent patients. Tumor capsule disruption emerges as the most critical factor influencing recurrence. By incorporating pathological features and surgical factors, we developed a recurrence prediction model that offers a more accurate risk assessment. These findings enhance our understanding of the disease trajectory and provide actionable insights for improving recurrence risk assessment and fertility preservation strategies in clinical practice.

## Figures and Tables

**Table 1:** Forms of recurrence.

Forms	FSS Group (54)		RS Group (14)	
	*n*	%	*n*	%
Imaging recurrence	52	96.3	14	100
Biochemical recurrence	29	53.7	13	92.9
Clinical recurrence	8	14.8	2	14.3

**Table 2:** Locations of recurrence.

Locations	FSS Group (54)		Locations	RS Group (14)	
	*n*	%		*n*	%
Ovary	47	87.0	Pelvic cavity	10	71.4
Ovary + pelvic cavity	2	3.7	Abdominal cavity	3	21.4
Ovary + abdominal cavity	1	1.9	Pelvic + abdominal cavity	1	7.1
Ovary + pelvic + abdominal cavity	2	3.7			
Pelvic cavity	2	3.7			

**Table 3:** Pathological consistency of 40 patients of FSS group before and after recurrence (*n* = 40).

Consistency	Before Recurrence	After Recurrence	*n*/%
Consistent			22/55.0
	classical SBOT		18/45.0
	MPSC		2/5.0
	SBOT with microinvasion		1/2.5
	microinvasive LGSOC		1/2.5
Inconsistent			18/45.0
Downgrade			9/22.5
	MPSC	classical SBOT	1/2.5
	SBOT with microinvasion	classical SBOT	6/15.0
	LGSOC	classical SBOT	1/2.5
	LGSOC	MPSC	1/2.5
Upgrade			9/22.5
	classical SBOT	MPSC	4/10.0
	classical SBOT	LGSOC	1/2.5
	SBOT with microinvasion	MPSC	2/5.0
	SBOT with microinvasion	LGSOC	1/2.5
	MPSC	LGSOC	1/2.5

**Table 4:** Pathological consistency of 8 patients of RS group before and after recurrence (*n* = 8).

Consistency	Before Recurrence	After Recurrence	*n*/%
Consistent			4/50.0
	classical SBOT		4/50.0
Inconsistent			4/50.0
Downgrade			1/12.5
	SBOT with microinvasion	classical SBOT	1/12.5
Upgrade			3/37.5
	classical SBOT	microinvasive LGSOC	1/12.5
	classical SBOT	LGSOC	1/12.5
	MPSC	LGSOC	1/12.5

**Table 5:** Clinicopathological characteristics of patients in FSS group (*n* = 66).

Factors	Recurrence Group *n* (%) or Median (Range)	Non-Recurrence Group *n* (%) or Median (Range)	*p*
Cases	54	12	
Age at diagnosis (y)			
≥40	5 (9.3)	3 (25.0)	0.152
<40	49 (90.7)	9 (75.0)	
CA125 (U/mL)	40.6 (9.3–1720.5)	37.4 (8.7–455.8)	0.470
Tumor size (cm)	7.6 (3.0–20.0)	6.8 (3.8–25.0)	0.790
Laterality			
Unilateral	32(59.3)	10 (83.3)	0.186
Bilateral	22(40.7)	2 (16.7)	
Tumor capsule disruption			
No	15 (27.8)	9 (75.0)	0.006 *
Yes	39 (72.2)	3 (25.0)	
RD			
No	49 (90.7)	12 (100.0)	0.575
Yes	5 (9.3)	0 (0)	
FIGO stage			
I	46 (85.2)	12 (100.0)	0.333
II–III	8 (14.8)	0 (0)	
Histology type			
Classical SBOT	33 (61.1)	11 (91.7)	0.462
MPSC	6 (11.1)	0 (0)	
SBOT with microinvasion	12(22.2)	1 (8.3)	
Microinvasive LGSOC	1 (1.9)	0 (0)	
LGSOC	2 (3.7)	0 (0)	
Micropapillary			
No	16 (29.6)	10 (83.3)	0.001 *
Yes	38 (70.4)	2 (16.7)	
Non-invasive implants			
No	46 (85.2)	12 (100.0)	0.333
Yes	8 (14.8)	0 (0)	
Invasive implants			
No	53 (98.1)	12 (100.0)	1
Yes	1 (1.9)	0 (0)	
Surgical approach			
Laparotomy	20 (37.0)	8 (66.7)	0.060
Laparoscopy	34 (63.0)	4 (33.3)	
FSS extent			
USO + CC	16 (29.6)	2 (16.7)	0.299
USO	15 (27.8)	6 (50.0)	
BC	14 (25.9)	1 (8.3)	
UC	9 (16.7)	3 (25.0)	
Complete staging			
Yes	9 (16.7)	3 (25.0)	0.679
No	45 (83.3)	9 (75.0)	
Adjuvant chemotherapy			
Yes	4 (7.4)	0 (0)	1
No	50 (92.6)	12 (100.0)	

* *p*-value < 0.05.

**Table 6:** Clinicopathological characteristics of patients in RS group (*n* = 52).

Factors	Recurrence Group *n* (%) or Median (range)	Non-Recurrence Group *n* (%) or Median (Range)	*p*
Cases	14	38	
Age at diagnosis (y)			
≥40	11 (78.6)	35 (92.1)	0.325
<40	3 (21.4)	3 (7.9)	
CA125 (U/mL)	57.1 (12.4–579.2)	55.4 (4.5–1096.03)	0.798
Tumor size (cm)	8.3 (4.0–15.0)	8.1 (3.2–19.1)	0.963
Laterality			
Unilateral	5 (35.7)	21 (55.3)	0.211
Bilateral	9(64.3)	17 (44.7)	
Tumor capsule disruption			
No	1 (7.1)	15 (39.5)	0.040 *
Yes	13 (92.9)	23 (60.5)	
RD			
No	14 (100)	36 (94.7)	0.545
Yes	0 (0)	2 (5.3)	
FIGO stage			
I	5 (35.7)	30 (78.9)	0.006 *
II–III	9(64.3)	8 (21.1)	
Histology type			
Classical SBOT	7 (50.0)	22 (57.9)	0.027 *
MPSC	2 (14.3)	7 (18.4)	
SBOT with microinvasion	3 (21.4)	5 (13.2)	
Microinvasive LGSOC	0 (0)	3 (7.9)	
LGSOC	2 (14.3)	1 (2.6)	
Micropapillary			
No	1 (7.1)	17 (44.7)	0.019 *
Yes	13 (92.9)	21 (55.3)	
Non-invasive implants			
No	5 (35.7)	31 (71.6)	0.005 *
Yes	9(64.3)	7 (18.4)	
Invasive implants			
No	14 (100)	36 (94.7)	1
Yes	0 (0)	2 (5.3)	
Surgical approach			
Laparotomy	8 (57.1)	25 (65.8)	0.566
Laparoscopy	6 (42.9)	13 (34.2)	
Complete staging			
Yes	12 (85.7)	30 (78.9)	0.710
No	2 (14.3)	8 (21.1)	
Adjuvant chemotherapy			
Yes	6 (42.9)	8 (21.1)	0.161
No	8 (57.1)	30 (78.9)	

* *p*-value < 0.05.

**Table 7:** Univariate and multivariate logistic regression analysis for recurrence in FSS group.

Variables	Univariate			Multivariate		
	Z	*p*	OR (95%CI)	Z	*p*	OR (95%CI)
Age at diagnosis (y)						
≥40						
<40	1.452	0.147	3.267 (0.661–16.150)			
Laterality						
Unilateral						
Bilateral	1.501	0.133	3.437 (0.686–17.237)			
Tumor capsule disruption						
No						
Yes	2.804	0.005 *	7.800 (1.856–32.788)	3.056	0.002 *	39.857 (3.751–423.493)
RD						
No						
Yes	0.009	0.993	10,419,137.716 (0.000–Inf)			
FIGO stage						
I						
II–III	0.007	0.994	30,169,250.418 (0.000–Inf)			
Histology type						
SBOT						
LGSOC	0.009	0.993	3,611,852.489 (0.000–Inf)			
Micropapillary						
No						
Yes	2.982	0.003 *	11.875 (2.335–60.403)	3.198	0.001 *	56.615 (4.769–672.061)
Non-invasive implants						
No						
Yes	0.007	0.994	30,169,250.418 (0.000–Inf)			
Invasive implants						
No						
Yes	0.010	0.992	1,303,655.742 (0.000–Inf)			
Surgical approach						
Laparotomy						
Laparoscopy	1.815	0.069	3.400 (0.907–12.743)			
Complete staging						
Yes						
No	0.672	0.502	1.667 (0.376–7.394)			
Adjuvant chemotherapy						
Yes						
No	−0.008	0.993	0.000 (0.000–Inf)			
CA125 (U/mL)	0.798	0.425	1.002 (0.997–1.006)			
Tumor size (cm)	−0.508	0.611	0.965 (0.841–1.108)			

* *p*-value < 0.05.

**Table 8:** Univariate and multivariate logistic regression analysis for recurrence in RS group.

Variables	Univariate			Multivariate		
	Z	*p*	OR (95%CI)	Z	*p*	OR (95%CI)
Age at diagnosis (y)						
≥40						
<40	1.305	0.192	3.182 (0.560–18.088)			
Laterality						
Unilateral						
Bilateral	1.237	0.216	2.224 (0.627–7.890)			
Tumor capsule disruption						
No						
Yes	1.962	0.050	8.478 (1.002–71.731)			
RD						
No						
Yes	−0.009	0.993	0.000 (0.000–Inf)			
FIGO stage						
I						
II–III	0.257	0.797	1.385 (0.116–16.581)			
Histology type						
SBOT						
LGSOC	1.434	0.152	6.167 (0.513–74.169)			
Micropapillary						
No						
Yes	2.164	0.030 *	10.524 (1.248–88.739)			
Non-invasive implants						
No						
Yes	2.977	0.003 *	7.971 (2.032–31.266)	2.977	0.003 *	7.971 (2.032–31.266)
Invasive implants						
No						
Yes	−0.009	0.993	0.000 (0.000–Inf)			
Surgical approach						
Laparotomy						
Laparoscopy	0.573	0.567	1.442 (0.412–5.048)			
Complete staging						
Yes						
No	−0.546	0.585	0.625 (0.116–3.380)			
Adjuvant chemotherapy						
Yes						
No	−1.542	0.123	0.356 (0.095–1.324)			
CA125 (U/mL)	−0.190	0.849	1.000 (0.997–1.002)			
Tumor size (cm)	−0.331	0.740	0.976 (0.844–1.128)			

* *p*-value < 0.05.

**Table 9:** Univariate and multivariate Cox proportional hazards regression analysis for recurrence in FSS group.

Variables	Univariate			Multivariate		
	Z	*p*	HR (95%CI)	Z	*p*	HR (95%CI)
Age at diagnosis (y)						
≥40						
<40	2.047	0.041 *	2.929 (1.047–8.194)			
Laterality						
Unilateral						
Bilateral	2.726	0.006 *	2.181 (1.245–3.821)	2.536	0.011 *	2.075 (1.180–3.648)
Tumor capsule disruption						
No						
Yes	2.855	0.004 *	2.462 (1.326–4.571)	2.718	0.007 *	2.369 (1.272–4.413)
RD						
No						
Yes	2.295	0.022 *	3.107 (1.180–8.180)			
FIGO stage						
I						
II–III	3.176	0.001 *	3.613 (1.635–7.983)			
Histology type						
SBOT						
LGSOC	0.979	0.328	2.047 (0.488–8.593)			
Micropapillary						
No						
Yes	1.289	0.197	1.478 (0.816–2.678)			
Non-invasive implants						
No						
Yes	2.874	0.004 *	3.174 (1.444–6.979)			
Invasive implants						
No						
Yes	1.176	0.240	3.354 (0.446–25.210)			
Surgical approach						
Laparotomy						
Laparoscopy	1.169	0.243	1.394 (0.799–2.433)			
Complete staging						
Yes						
No	−0.365	0.715	0.874 (0.423–1.805)			
Adjuvant chemotherapy						
Yes						
No	−2.327	0.020 *	0.281 (0.097–0.819)			
CA125 (U/mL)	1.741	0.082	1.001 (1.000–1.002)			
Tumor size (cm)	0.781	0.435	1.024 (0.965–1.086)			

* *p*-value < 0.05.

**Table 10:** Univariate and multivariate Cox proportional hazards regression analysis for recurrence in RS group.

Variables	Univariate			Multivariate		
	Z	*p*	HR (95%CI)	Z	*p*	HR (95%CI)
Age at diagnosis (y)						
≥40						
<40	1.303	0.192	2.364 (0.648–8.616)			
Laterality						
Unilateral						
Bilateral	1.253	0.210	2.021 (0.673–6.074)			
Tumor capsule disruption						
No						
Yes	1.719	0.086	5.973 (0.778–45.838)			
RD						
No						
Yes	−0.002	0.998	0.000 (0.000–Inf)			
FIGO stage						
I						
II–III	2.805	0.005 *	4.870 (1.611–14.721)			
Histology type						
SBOT						
LGSOC	2.259	0.024 *	6.022 (1.268–28.606)	3.128	0.002 *	19.809 (3.050–128.648)
Micropapillary						
No						
Yes	2.251	0.024 *	10.530 (1.356–81.802)			
Non-invasive implants						
No						
Yes	2.971	0.003 *	5.326 (1.767–16.054)	3.237	0.001 *	8.840 (2.363–33.075)
Invasive implants						
No						
Yes	−0.002	0.998	0.000 (0.000–Inf)			
Surgical approach						
Laparotomy						
Laparoscopy	0.030	0.976	1.016 (0.349–2.964)			
Complete staging						
Yes						
No	−1.215	0.224	0.384 (0.082–1.799)			
Adjuvant chemotherapy						
Yes						
No	−1.713	0.087	0.391 (0.134–1.145)			
CA125 (U/mL)	0.274	0.784	1.000 (0.998–1.003)			
Tumor size (cm	0.368	0.713	1.025 (0.897–1.172)			

* *p*-value < 0.05.

## Data Availability

Anonymized data can be made available by contacting the senior author.

## Abbreviations

The following abbreviations are used in this manuscript:

SBOT	Serous borderline ovarian tumors
LGSOC	Low-grade serous ovarian carcinoma
RFS	Recurrence-free survival
C-index	Concordance index
AUC	Area under the curve
ROC	Receiver operating characteristic
DCA	Decision curve analysis
FSS	Fertility-sparing surgery
CI	Confidence interval
EOC	Epithelial ovarian cancer
WHO	World Health Organization
HGSOC	High-grade serous ovarian carcinoma
BOT	Borderline ovarian tumors
MPSC	Micropapillary serous carcinoma
FIGO	International Federation of Gynecology and Obstetrics
RD	Gross residual disease
NCCN	National Comprehensive Cancer Network
WHO	World Health Organization
UC	Unilateral ovarian cystectomy
BC	Bilateral ovarian cystectomy
USO	Unilateral salpingo-oophorectomy
USO + CC	Unilateral salpingo-oophorectomy and contralateral ovarian cystectomy
TH + BSO	Total hysterectomy and bilateral salpingo-oophorectomy
CA125	Carbohydrate antigen 125
SD	Standard deviation
